# Case Report: Gastrointestinal PEComa With *TFE3* Rearrangement Treated With Anti-VEGFR TKI Apatinib

**DOI:** 10.3389/fonc.2020.582087

**Published:** 2020-11-23

**Authors:** Jiaming Xu, Xiao-Lei Gong, Huanwen Wu, Lin Zhao

**Affiliations:** ^1^ Department of Medical Oncology, Peking Union Medical College Hospital, Chinese Academy of Medical Sciences and Peking Union Medical College, Beijing, China; ^2^ Department of Pathology, Peking Union Medical College Hospital, Chinese Academy of Medical Sciences and Peking Union Medical College, Beijing, China

**Keywords:** perivascular epithelioid cell tumors, *TFE3*, mammalian target of rapamycin inhibitor, anti-VEGFR, tyrosine kinase inhibitor, apatinib

## Abstract

Perivascular epithelioid cell tumors (PEComas) are rare mesenchymal tumors. Unresectable malignant PEComas with *TFE3* rearrangement have no recommended therapy to date. Here, we report the first case of malignant gastrointestinal PEComa with *TFE3* rearrangement which has a response to the targeted therapy of an anti-VEGFR tyrosine kinase inhibitor (TKI), apatinib. A 31-year-old female was diagnosed with malignant gastrointestinal PEComa with *TFE3* rearrangement and hepatic metastases. A resection of the giant retroperitoneal mass was performed. The patient received the anti-VEGFR TKI apatinib to treat the hepatic metastasis. The tumor remained stable during apatinib treatment and the progression-free survival (PFS) lasted about 7 months. This case suggests that targeting the VEGF/VEGFR signaling pathway may be an essential new therapeutic choice for *TFE3*-associated malignant PEComas.

## Introduction

Perivascular epithelioid cell tumors (PEComas) are a group of rare mesenchymal tumors. They are composed of perivascular epithelioid cells (PECs) with distinctive histological and immunohistochemical characteristics (1). PECs are usually epithelioid or spindle-shaped, expressing both melanocytic and muscle markers (2). The PEComa family now includes angiomyolipoma (AML), clear cell “sugar” tumor (CCST) of the lung, lymphangioleiomyomatosis (LAM), and other neoplasms with aforementioned histological and immunohistochemical characteristics at various anatomical sites (3). AML and LAM are strongly associated with tuberous sclerosis complex (TSC), but another non-classic subset of PEComas associated with transcription factor E3 (*TFE3*) gene rearrangement has been reported in various sites of human body (2). Generally PEComas have a benign behavior but malignant cases have been reported at different sites (4). PEComas are usually treated with surgery (5). As an option of target therapy, mammalian target of rapamycin (mTOR) inhibitors have been proved safe and effective in the treatment of unresectable PEComas associated with TSC (6). However, mTOR inhibitors may lose effect in PEComas with *TFE3* gene rearrangement (7), which calls for the investigation of more specific and effective therapies. Here we report a malignant case of PEComa in the gastrointestinal tract with hepatic metastasis and *TFE3* gene rearrangement, which had a response to the treatment of the anti-VEGFR tyrosine kinase inhibitor (TKI), apatinib.

## Case Description

A 31-year-old woman presented with 3-month history of increased abdominal circumference and intermittent fever. The patient complained no other discomforts. She also denied any anomalies in the past and in the family history. In February 2018 the computed tomography (CT) scans showed a large mass in the upper left abdominal cavity. The mass measured about 16.8×12.5cm, receiving blood supply from celiac trunk, splenic artery and branches of left gastric artery. Splenic vein drained blood flow of the tumor into portal vein. Other abnormalities include retroperitoneal lymphadenopathy, multiple hepatic metastases, enlarged left adrenal gland, and multiple small lymph nodes in the mesentery, pelvis and bilateral groin regions. The somatostatin receptor imaging and tomography showed increased expression of somatostatin receptor with necrosis in the left upper abdominal cavity and multiple hepatic space-occupying lesions.

**Figure 1:**
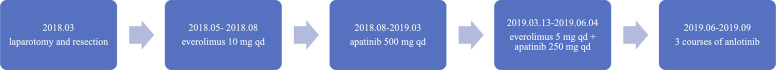
Timeline with relevant data from the episode of medical care.

**Figure 2:**
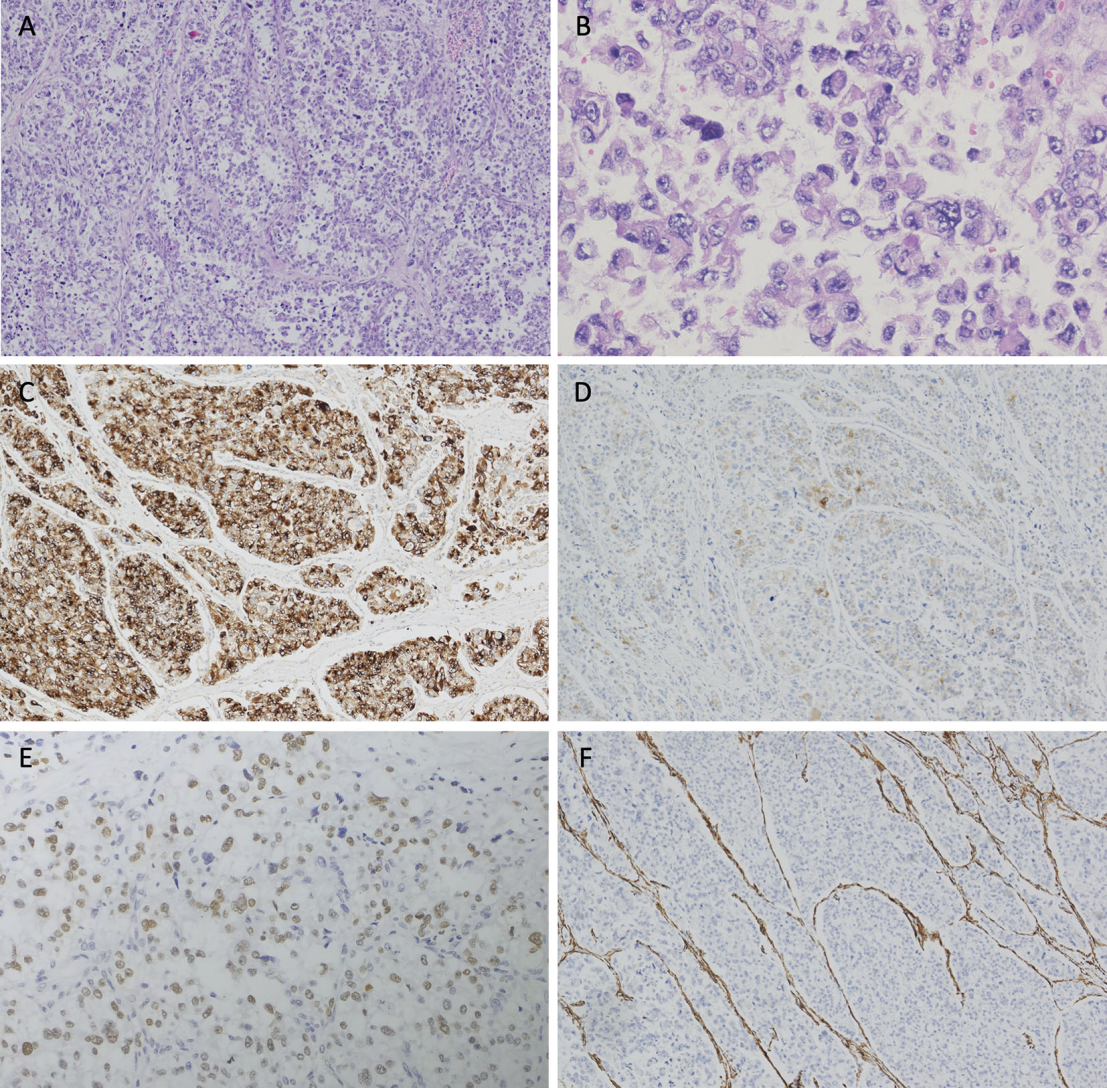
Hematoxylin and eosin (H&E) staining and immunohistochemical analyses of the resected tumor. **(A)** The tumor cells arrange in a diffuse pattern(×4). **(B)** Epithelioid tumor cells are oval or polygonal, with clear or granular eosinophilic cytoplasm. Nucleoli are prominent in the vesicular nuclei. Increased mitotic activity and dysplasia are observed (×20). Tumor cells are positive for **(C)** HMB-45 (strong and diffuse), **(D)** Melan-A (patchy) and **(E)** *TFE3* (modest and diffuse), and are negative for **(F)** SMA.

**Figure 3:**
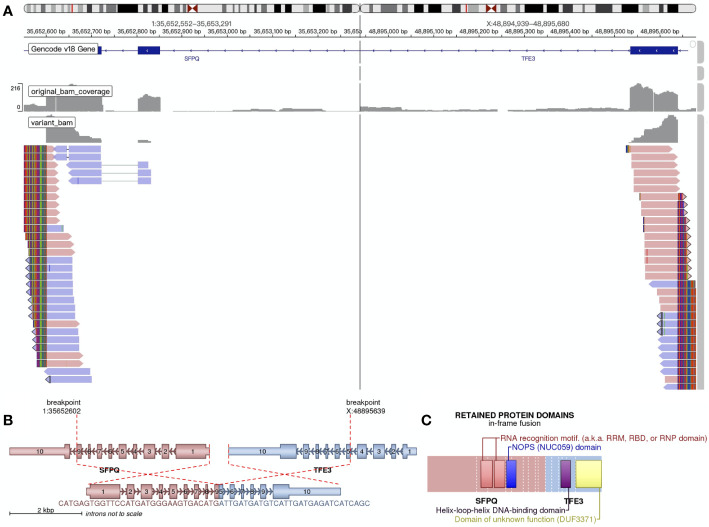
RNA-based next-generation sequencing identified SFPQ-*TFE3* fusion in tumor cells. **(A)** Visualization of the sequencing results through the Integrative Genomics Viewer (IGV). A SFPQ-*TFE3* fusion was showed. **(B)** shows the location where the fusion took place. **(C)** shows the retained protein domains of the fusion protein.

A timeline of the episode of medical care is shown in **Figure 1**. A laparotomy and resection of the giant retroperitoneal mass (including partial posterior gastric wall, spleen, pancreatic body and tail, and partial left adrenal gland) was performed in March 2018. During surgery the mass was found located in the omental sac, closely associated with posterior gastric wall and was about 25×25×20 cm. The mass disseminated widely in the abdominal cavity. Pathological findings revealed malignant PEComa of the gastrointestinal tract with diffuse expression of TFE3. Invasion of gastric mucosa and muscularis and pancreas with necrosis were noted. Lymph nodes showed chronic inflammation. Immunohistochemical stains (**Figure 2**) showed tumor cells positive for Melan-A (patchy), HMB-45 (strong and diffuse), vimentin (patchy) and *TFE3* (modest and diffuse), and negative for PAX-8, S-100, Syn, CK7, desmin, Myo-D1, SMA, and calponin. Ki67 labeling index in the tumor cells was 30%. RNA-based next-generation sequencing identified SFPQ-*TFE3* fusion in tumor cells (**Figure 3**). A diagnosis of advanced malignant PEComa of the gastrointestinal tract with *TFE3* rearrangement and hepatic metastases was made.

**Figure 4:**
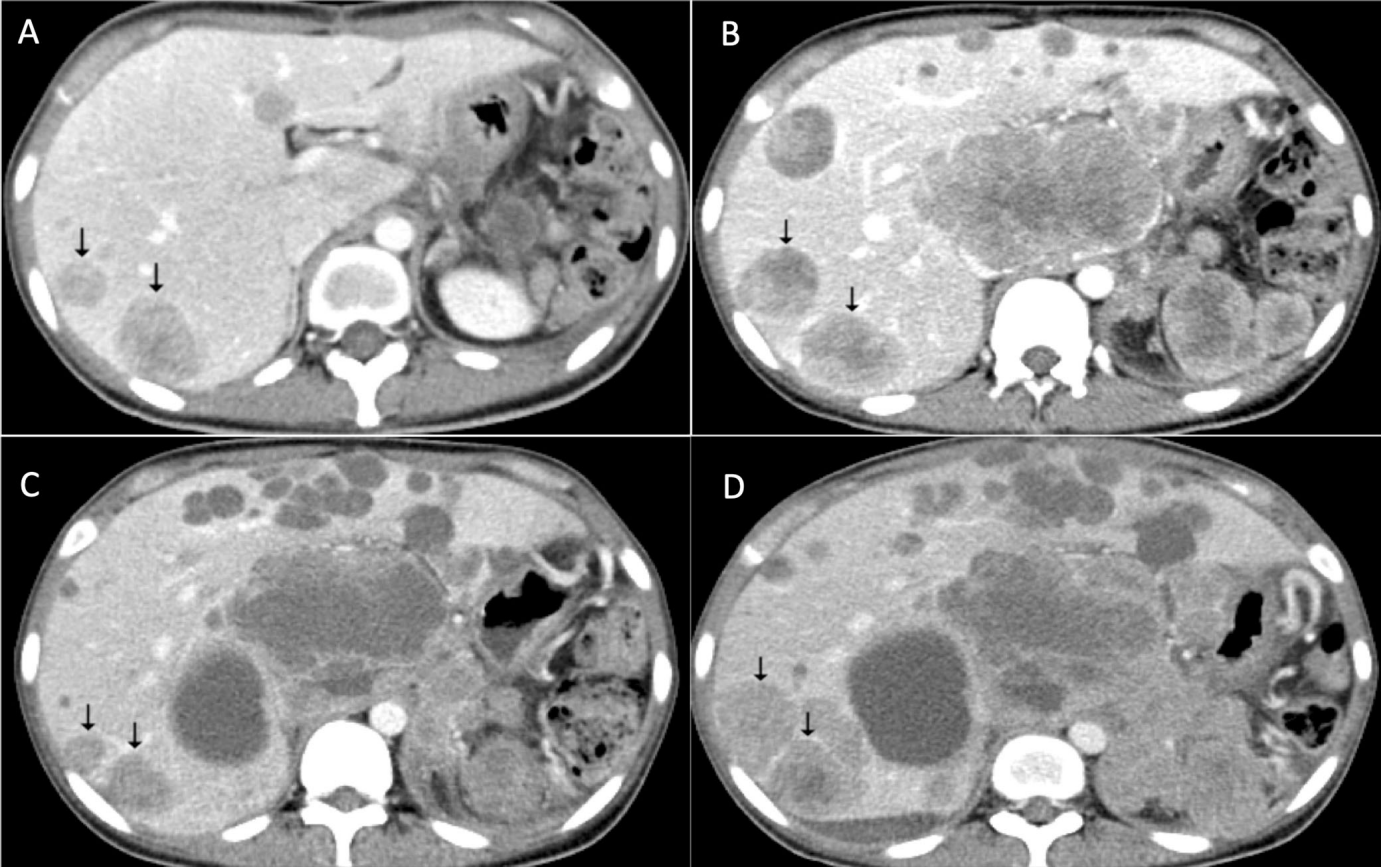
Response of apatinib for metastatic PEComa of the liver. **(A)** Multiple hepatic metastases revealed after surgery. **(B)** Tumor progression after everolimus treatment. **(C)** Response of hepatic metastases after apatinib treatment. **(D)** Tumor progression in April 2019.

Unfortunately, the postoperative imaging showed progression of hepatic metastases (**Figure 4A**) and systemic treatment was needed. However, no effective therapy of PEComas with *TFE3* expression was reported. Therefore, we decided to first use the mTOR inhibitor everolimus and see whether it worked on the patient. From May 6th she began to take mTOR inhibitor everolimus 10 mg qd as first-line treatment for 3 months. Restaging CT showed tumor progression on everolimus treatment (**Figure 4B**). Considering the gastrointestinal origin of the hepatic metastases, from August 13^th^ she was treated with 500 mg qd apatinib, which is an anti-VEGFR2 TKI used in third-line treatment of gastric cancer (GC) in China. The tumor remained stable (but with necrosis in the center, **Figure 4C**) and the progression-free survival (PFS) lasted about 7 months. During apatinib treatment the patient reported a remission of abdominal bloating. Mild proteinuria was confirmed through the routine urine test, and no other adverse events were observed. In March 2019, the hepatic metastases progressed (**Figure 4D**). In addition, multiple metastases of the tumor cells were detected in the right lung, greater omentum and pelvic cavity. Therefore we decided to try the combination therapy. From March 13^th^ 2019 the combination targeted therapy of everolimus 5 mg qd and apatinib 250 mg qd began, but no obvious improvement was observed. On June 4^th^ she stopped taking everolimus due to worsening cough, which might result from repeated pulmonary infections. From June 18th, she received anlotinib treatment for three courses with 2 weeks of medication and 1 week off. Unfortunately, the tumor progressed and the patient passed away in September 2019.

## Discussion

In 1992 Bonetti et al introduced the term “perivascular epithelioid cell (PEC)” to identify a novel cell type characterized by immunoreactivity with melanocyte markers, an epithelioid appearance, a clear or granular eosinophilic cytoplasm, and a perivascular distribution (8). In 1996 Zamboni et al. suggested the term PEComa for neoplasms totally composed of proliferation of PECs (9). The most reported cases of PEComas happened in the lung, liver and kidney. Gastrointestinal PEComas account for 20% to 25% of the rest reported cases in human body. The malignant rate of gastrointestinal PEComas is relatively high (up to 52%), compared with those reported in other regions (for example, in the head and neck region the malignant rate is about 15.4%) (10, 11). The patients may have various symptoms, including abdominal pain, obstruction, anemia, depending on tumor size and organs involved (10). In 2005, Folpe et al. (4) proposed the criteria for PEComas with malignant characteristics. Neoplasms are considered malignant with 2 or more following histologic features: size > 5 cm, infiltrative, high nuclear grade and cellularity, mitotic rate ≥ 1/50 HPF, necrosis and vascular invasion. In our case the mass measured about 25×25×20 cm, with infiltrative growth and necrosis in the gastric wall and pancreas as well as hepatic metastases. In addition, Ki67 labeling index in the tumor cells was 30%. Therefore it was considered malignant.

PEComas express both melanocytic markers (such as HMB-45, Melan-A, tyrosinase, and MiTF) and muscle markers (such as SMA, muscle myosin, and calponin) (2). Occasionally positive cases of desmin, cytokeratin, S-100, and CD1a are reported (2). The most sensitive melanocytic markers for the diagnosis of PEComas are HMB-45 and Melan-A (12). Our case demonstrated that tumors cell positive for HMB-45 and Melan-A, and negative for desmin, SMA, and S-100. Therefore, the diagnosis of PEComa was made.

Some PEComas are strongly associated with tuberous sclerosis complex (TSC) (2). The mutant genes in TSC are *TSC1* and *TSC2*, whose gene products play a role in inhibiting mTOR. mTOR regulates cell division, therefore its hyperactivation contributes to TSC, resulting from deficiency of *TSC1* and *TSC2* (13). This mechanism indicates that mTOR inhibitor may serve as a therapy for TSC and TSC-related PEComas. Evidence has shown the efficacy of mTOR inhibitors, with partial or complete response in patients with TSC-related PEComas (6 of 11) (14). About 10% of PEComas are associated with *TFE3* rearrangement (4). These include tumors with SFPQ/PSF-*TFE3* fusion or DVL2-*TFE3* fusion (15), which are often observed in young patients without TSC2 alternations (7, 16). Different from the reported cases using FISH to detect the *TFE3* rearrangement (11, 17), we conducted RNA-based next-generation sequencing technique to directly position the fusion locus, and found a SFPQ-*TFE3* fusion in the tumor cells. A recent study suggests that PEComas with *TFE3* rearrangement be reclassified as “melanotic Xp11 neoplasm” or “Xp11 neoplasm with melanocytic differentiation”, because clinicopathologic data and outcome analysis indicate that these tumors have a closer relationship with alveolar soft part sarcomas (ASPS) instead of conventional PEComas with TSC mutations (18). Therefore, *TFE3*-rearranged PEComas may hypothetically be nonresponsive to mTOR inhibitor therapy (19). Our patients received mTOR inhibitor everolimus. However, the first-line treatment showed no response to everolimus and tumor progression, which appeared to support the inefficacy of mTOR inhibitors in PEComas with *TFE3* rearrangement.

The inefficacy of mTOR inhibitors in our case urged us to find a better therapy. Here we turned to VEGFR inhibitors. It was reported that a rise in serum VEGF-D concentration is often observed in PEComa patients, which is associated with clinical presentation of PEComa (20), suggesting that targeting the VEGF/VEGFR signaling pathway should be a potential treatment for malignant PEComas. An attenuation of VEGF-D upregulation in serum and inhibition of LAM is observed in a mouse model treated with VEGFR inhibitor axitinib (21). In our case we are the first to report the therapy of VEGFR-2 inhibitor apatinib to treat PEComa with *TFE3* rearrangement. Apatinib is a newly developed anti-angiogenetic agent in China used in third-line treatment of GC, which is an anti-VEGFR2 TKI and has shown efficacy in GC, hepatocellular carcinoma (HCC), and non-small cell lung cancer (NSCLC) (22). The anti-angiogenetic effect of apatinib may help to normalize tumor angiogenesis and to enhance the sensitivity of chemotherapy (23). After receiving apatinib treatment the tumor remained stable for 7 months without progression, which showed that the anti-VGEFR effect might be useful in the treatment of malignant PEComas. Generally, the adverse reactions of apatinib treatment are associated with hypertension, urine protein, hand foot syndrome and gastrointestinal reaction (diarrhea) (22). In our case, mild proteinuria was confirmed in the patient, while hypertension was not observed.

When the tumor finally progressed, we chose the combination therapy of the anti-VEGFR TKI apatinib and an mTOR inhibitor everolimus. However, the outcome was not satisfactory and the tumor kept on progressing. By contrast, a recent study reported that a patient with malignant uterine PEComa receiving combination targeted therapy of a VEGFR inhibitor (sorafenib) with an mTOR inhibitor (sirolimus) has shown a complete response (24). Therefore, whether the combination targeted therapy is useful in *TFE3*-associated malignant PEComas needs more investigation.

Recently the largest international retrospective series analyzed the therapeutic effects of VEGFR inhibitors (pazopanib, sorafenib, and sunitinib) in advanced PEComas. In the retrospective series, the ORR for VEGFR inhibitors was 8.3%, and the median PFS was 5.4 months (25). However, the limitation of the reported VEGFR inhibitors therapy was that the authors did not mention the mutational status of the patients. In other words, we still don’t know whether the therapeutic effects of anti-VEGFR TKI are different between TSC1/2-mutated and TFE3-associated PEComas. By contrast, our case specifically shows the therapeutic potential of anti-VEGFR TKI in *TFE3*-associated PEComas. We consider that it’s an innovative perspective.

Another recent retrospective series reported the reversion of the resistance to mTOR inhibitors by adding exemestane in advanced PEComas (with a median PFS of 7 months and a median DOR of 11 months) (26), which indicated that the combination of mTOR inhibitors and exemestane would be effective after progression on mTOR inhibitor monotherapy. However, the combination therapy of mTOR inhibitors and exemestane were effective only in TSC1/2-mutated PEComas, whether it has effect in *TFE3*-associated PEComas still needs more exploration. By contrast, in this case report the anti-VEGFR TKI apatinib directly took effect in advanced PEComas with *TFE3* rearrangement, which suggested a better therapy for *TFE3*-associated PEComas than the combination of mTOR inhibitors and exemestane.

Through our exploration, we consider that the anti-VEGFR effect may play a more important role in the treatment of PEComa with *TFE3* rearrangement. Therefore, targeting the VEGF/VEGFR signaling pathway may be a useful strategy for unresectable PEComas, especially those with *TFE3* rearrangement and a limited response to mTOR inhibitors, but to what extent the tumor can be restrained needs more investigation. Future studies should explore the mechanism of apatinib inducing the clinical effects, and whether the combination therapy is useful in *TFE3*-associated malignant PEComas.

## Conclusions

For malignant PEComas with *TFE3* rearrangement, the mTOR inhibitor therapy shows limited effect, calling for new therapies. One of the strategies is to target another VEGF/VEGFR signaling pathway. Here we are the first to report that VEGFR inhibitor apatinib had a 7 months duration of response in a patient with advanced malignant PEComa with *TFE3* rearrangement. This case suggests that targeting the VEGF/VEGFR signaling pathway may be an essential new therapeutic choice for *TFE3*-associated malignant PEComas.

## Data Availability Statement

The original contributions presented in the study are included in the article/supplementary material. Further inquiries can be directed to the corresponding author.

## Ethics Statement

Informed consent was obtained from the patient for publication of this case report and any accompanying images. The patient gave consent to publish this case report, and read the article and confirmed its content.

## Author Contributions

JX, X-LG and LZ designed the study, conducted the literature review, collected the data and wrote and revised the draft manuscript and subsequent manuscripts. HW conducted the experiments. JX and X-LG contributed equally to the manuscript. All authors contributed to the article and approved the submitted version.

## Conflict of Interest

The authors declare that the research was conducted in the absence of any commercial or financial relationships that could be construed as a potential conflict of interest.
